# Futile Sexual Homicide in Nepal: A Case Report

**DOI:** 10.31729/jnma.6057

**Published:** 2021-11-30

**Authors:** Alok Atreya, Shiva Pandit, Samata Nepal, Jun Bajracharaya, Deepak Shrestha

**Affiliations:** 1 Department of Forensic Medicine, Lumbini Medical College, Palpa, Nepal; 2 Department of Otorhinolaryngology, Lumbini Medical College, Palpa, Nepal; 3 Department of Community Medicine, Lumbini Medical College, Palpa, Nepal; 4 Lumbini Medical College, Palpa, Nepal; 5 Department of Obstetrics and Gynaecology, Lumbini Medical College, Palpa, Nepal

**Keywords:** *child abuse*, *sexual*, *homicide*, *Nepal*, *rape*, *sex offenses*

## Abstract

Although cases of sexual offenses are not uncommon in children, they present to the Emergency Department seeking treatment for a medical cause. Sometimes the history of abuse is missed by the treating clinicians who are only focused upon the presenting complaint and not upon the underlying cause. Furthermore, the lack of reporting of sexual abuse in medical literatures makes them a rarity in the Nepalese scenario. We present an uncommon case of a child where the perpetrator who tried to silence her during the sexual intercourse made a futile attempt to kill her cutting her throat with a sickle.

## INTRODUCTION

Sexual homicide comprises of two key elements; homicide of the victim and sexual behaviour of the perpetrator.^1^ The rebuttal of the victim to satisfy the immediate sexual desire of the offender may trigger the wrathful homicide.^2^ Although cases of sexual offenses are not uncommon, the lack of reporting makes them a rarity in the Nepalese scenario. We present an uncommon case of a child where the perpetrator who tried to silence her during the sexual intercourse made a futile attempt to kill her cutting her throat with a sickle.

## CASE REPORT

A 4-year-old girl was returning home from school when she was intercepted by a 16-year-old boy from the same neighbourhood. The incident took place at around 2 pm in the afternoon on the clay mountainous road which ran through the woods. The boy who was cutting cattle fodder and collecting firewood happened to be her senior from the eighth grade from the same school she attended. As per the narrative described, the girl was first grabbed by the hips and then her skirt was lifted to which the girl retaliated by screaming. The boy had told the girl to keep quiet to which the girl didn't listen to. The boy then furiously cut her throat with his sickle lying nearby and fled the scene. The girl then ran towards her home where she was rescued and rushed to the nearby primary health care centre (PHC). After the initial assessment and the medicolegal nature of the case, she was referred to a tertiary care centre 110 kilometres away.

**Figure 1:**
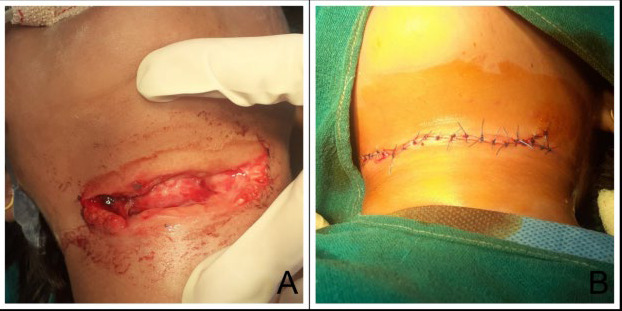
(A) Horizontally placed incised wound at the front of the neck at the level of thyroid cartilage; (B) the wound after surgical repair.

When presented to the Emergency Department (ED) of Lumbini Medical College Teaching Hospital, Palpa with a cut-throat injury at around 10 o'clock in the evening, the patient was conscious but anxious. Her vitals were stable and within normal limits. There was no shortness of breath and the speech was intact. On local examination an incised wound was present at the front of the neck without any active bleeding. Carotid pulse was palpable on both the sides. General and systemic examination revealed no significant findings. Computed tomography of the cervical spine showed multiple foci of air density within deep neck spaces including the pharyngeal mucosal spaces, parapharyngeal, right carotid, right parathyroid and retro pharyngeal spaces. There were multiple foci of air density at para tracheal soft tissue as well as prevertebral soft tissue extending into superior mediastinum. The patient was then shifted to the operation theatre (OT) where the wound was explored under general anaesthesia. A horizontally placed incised wound measuring 9.5 × 1 cm, was present on the neck in midline exposing the thyroid cartilage through the gaping (Figure 1). Wound was deeper on the right side, with regular and clean-cut margins without any tentative cuts. The wound was cleaned and washed with normal saline solution and the clots removed. The pharynx and vital neck structures were intact. The wound was closed in layers.

The gynaecological examination revealed no marks of violence on the genitals of the child. The labia majora were in close apposition even after complete abduction of the thighs. Retraction of the labia revealed an intact hymen. No sign of struggle was present over the body.

## DISCUSSION

Child victims of sexual abuse frequently present to the ED with a medical complaint rather than with a history of abuse.^3^ In the present case the victim presented to ED with a cut throat injury at the level of the thyroid. The wound was situated on both the sides, was transverse without any tailing and hesitation cuts pointing positively towards the homicidal nature. The absence of defence wound and signs of struggle in the victim suggested that the victim did not know what was happening and also was incapable of resisting. However, victim-blaming still persists in the society and it has been viewed that child victims themselves are responsible for victimization if they failed to resist sexual advances in the first place.^4,5^ A study on child sexual abuse found that the victim's age and her relation to the perpetrator could influence her response to abuse as encouraging, passive or resisting; the perception of opinion also varied with these characteristics.^6^

The perpetrator tried to kill the victim slitting her throat, but the victim survived. Any forceful attempt of sexual intercourse in a pre-pubertal girl, it is more likely that the prominent tissues of the labia majora can be contused, abraded, and lacerated particularly on the anterior half. The absence of a mark of violence in the present case rules out penetration. As per the Muluki Criminal Code of Nepal, a mere touch of the male genital in the female vulva is sufficient to constitute rape.^7^

A 23-year study (1994 to 2016) on sexual homicide from China revealed that the majority of the offenses were heterosexual committed mostly by male perpetrators during the day indoors.^8^ The same study showed edged weapon was used only in 28.8% of cases.

An exploratory and descriptive study in France upon sexual homicide convicts, where the data was collected from 46 courts between the years 1975 and 2012, showed all the perpetrators were males, 83.3% of who were sexually excited during the crime phase.^9^ The majority of the victims in the study were female victims aged <14 years of age.^9^ In almost all cases of sexual violence, the victims are coerced to comply with the demands of the perpetrator where the perpetrator uses verbal threats, aggression, or abusive language. If the victims did not comply, the perpetrator became raged with increased violence intensity, often killing the victim.^8-10^ In the present case it was the cut-throat injury inflicted upon the victim.

The perpetrator who could not control his immediate sexual need will be punished by the law; but the innocent child who did not know the nature and severity of the act, will have to live the rest of her life with the scars. The justice system is stringent in Nepal when it comes to sexual offenses, however, the number of cases is still on the rise. Although the constitution of Nepal provides equal rights to all of its citizens irrespective of gender, the patriarchal society still considers females as an inferior member.^11^ The law has demarcated a fine line on 'what sex should not be' but not what sex should be. It is the cultural norm and societal brought up that has to change. One should be taught to value the women rights. Sex is not a battle over the women to be won. The patriarchal nature of the society does not intent to realize the consent of the woman to be vital when it comes to sexual intercourse which is a consensual act. Legally, even the marriage does not provide impunity for sexual intercourse by the husband without the wife's consent, and the husband can be charged with imprisonment and punishment for the offense of marital rape. Until all the males in the society uproot their traditional mindset of being superior to women and start considering them as equal, making their voices heard, violence upon women will not decline.

Cases of child sexual abuse come to ED with medical complaints and conditions which require immediate attention. They are reluctant to give a proper history of abuse due to hesitation as the patriarchal nature of our society regards sexual offences as a taboo. Furthermore, blaming the victim for not resisting, or blaming her for sexual advancement is still prevalent which further victimizes the victim. In the present case, otorhinolaryngologist who was involved in the emergency management of the patient consulted the forensic medicine department for the unnatural nature of the injury. The manner of the injury and the underlying cause was then revealed. A medico-legal case was filed, police was informed, and gynaecologist was added to the team for gynaecological examination. The history, findings and samples collected were handed over to the police for further investigation. The patient was provided with psychiatric counselling once she was stable. Young doctors of the ED are well versed with their medical duty, however, due to inexperience or ignorance, the legal duty they owe to the state is overlooked. It is the legal duty of the medical doctor to report the unnatural nature of the incident so that the victim would get legal remedy apart from the medical treatment. A multi-disciplinary approach in providing care for a patient is always beneficial to the patient and also minimizes the risk of medical error and litigation.

## References

[1] Karakasi MV, Vasilikos E, Voultsos P, Vlachaki A, Pavlidis P (2017). Sexual homicide: Brief review of the literature and case report involving rape, genital mutilation and human arson.. J Forensic Leg Med..

[2] Meloy JR (2000). The nature and dynamics of sexual homicide: An integrative review.. Aggress Violent Behav..

[3] Atreya A, Shrestha R, Nepal B, Nepal S, Shrestha D, Mahato S (2020). When sexual offence is an unexpected diagnosis - exploration of medical, legal and social aspects in Nepalese scenario.. Med Leg J..

[4] Back S, Lips HM (1998). Child sexual abuse: victim age, victim gender, and observer gender as factors contributing to attributions of responsibility.. Child Abuse Negl..

[5] Waterman CK, Foss-Goodman D (1984). Child molesting: Variables relating to attribution of fault to victims, offenders, and nonparticipating parents.. J Sex Res..

[6] Reynolds LL, Birkimer JC (2002). Perceptions of child sexual abuse:victim and perpetrator characteristics, treatment efficacy, and lay vs. legal opinions of abuse.. J Child Sex Abus..

[7] Nepal Law Commision. (2019). The Muluki Ain (General Code) [Internet]..

[8] Chan HCO, Li F, Liu S, Lu X, Jia H (2019). Sexual homicides in China: exploring the offender, victim, and offense characteristics.. Int J Offender Ther Comp Criminol..

[9] Higgs T, James J, Proulx J (2019). The Unusual Suspects: Multiple-Perpetrator and Multiple Concurrent Victim Sexual Homicide.. Int J Offender Ther Comp Criminol..

[10] Hamdi NR, Knight RA (2012). The relationships of perpetrator and victim substance use to the sexual aggression of rapists and child molesters.. Sex Abuse..

[11] Atreya A, Shrestha M, Acharya J, Gurung S (2019). Nepal - exploited by older married man - young unmarried mother accused of infanticide.. Med Leg J..

